# Knowledge, attitudes, beliefs and behaviours of older adults about pneumococcal immunization, a Public Health Agency of Canada/Canadian Institutes of Health Research Influenza Research Network (PCIRN) investigation

**DOI:** 10.1186/1471-2458-14-442

**Published:** 2014-05-12

**Authors:** Amy Schneeberg, Julie A Bettinger, Shelly McNeil, Brian J Ward, Marc Dionne, Curtis Cooper, Brenda Coleman, Mark Loeb, Ethan Rubinstein, Janet McElhaney, David W Scheifele, Scott A Halperin

**Affiliations:** 1 School of Population and Public Health, University of British Columbia, Vancouver, British Columbia, Canada; 2 Vaccine Evaluation Center, British Columbia Children’s Hospital and the University of British Columbia, Vancouver, British Columbia, Canada; 3 Canadian Center for Vaccinology, Dalhousie University, Capital Health and IWK Health Centre Halifax, Halifax, Nova Scotia, Canada; 4 Vaccine Study Centre, Research Institute of the McGill University Health Centre, Montreal, Quebec, Canada; 5 Research Center, Centre Hospitalier Universitaire, Quebec City, Quebec, Canada; 6 The Ottawa Hospital Research Institute, University of Ottawa, Ontario, Canada; 7 University of Toronto and Mount Sinai Hospital, Toronto, Ontario, Canada; 8 McMaster University, Hamilton, Ontario, Canada; 9 University of Manitoba, Department of Medicine, Winnipeg, Manitoba, Canada; 10 University of British Columbia, VITALiTY Research Center, Vancouver, British Columbia, Canada

**Keywords:** Invasive pneumococcal disease, Polysaccharide pneumococcal vaccine, Older adults

## Abstract

**Background:**

Fewer Canadian seniors are vaccinated against pneumococcal disease than receive the influenza vaccine annually. Improved understanding of factors influencing pneumococcal vaccination among older adults is needed to improve vaccine uptake.

**Methods:**

A self-administered survey measuring knowledge, attitudes, beliefs and behaviours about pneumococcal vaccination was administered to a cohort of seniors participating in a clinical trial of seasonal influenza vaccines at eight centers across Canada. Eligible participants were ambulatory adults 65 years of age or older, in good health or with stable health conditions, previously given influenza vaccine. The primary outcome was self-reported receipt of pneumococcal vaccination. Multi-variable logistic regression was used to determine factors significantly associated with pneumococcal vaccine receipt.

**Results:**

A total of 863 participants completed questionnaires (response rate 92%); 58% indicated they had received the pneumococcal vaccine. Being offered the vaccine by a health care provider had the strongest relationship with vaccine receipt (AOR 23.4 (95% CI 13.4-40.7)). Other variables that remained significantly associated with vaccine receipt in the multivariable model included having heard of the vaccine (AOR 10.1(95% CI 4.7-21.7)), and strongly agreeing that it is important for adults > 65 to be vaccinated against pneumococcus (AOR 3.3 (95% CI 1.2-9.2)). Participants who were < 70 years of age were less likely to be vaccinated.

**Conclusions:**

These results indicate healthcare recommendation significantly influenced vaccine uptake in this population of older adults. Measures to encourage healthcare providers to offer the vaccine may help increase coverage.

## Background

Invasive pneumococcal disease (IPD), caused by the *Streptococcus pneumoniae* bacterium, frequently results in serious outcomes including bacteremia, meningitis, bacterial pneumonia and death; vaccination is the primary means of prevention. In 2010, the incidence of IPD among adults 60 years of age and older in Canada was 23.2 per 100,000 and accounted for 48% of IPD cases reported in the country [1]. Vaccination coverage against pneumococcal disease is consistently lower than coverage for seasonal influenza among the elderly population in Canada and globally [2]. The most recent data available from the 2006 Canadian National Immunization Coverage Survey, based on self-report of vaccination status, estimated that only 39% of individuals 65 years of age and older had ever been vaccinated against pneumococcal disease whereas 70% had received the influenza vaccine that season [3]. A 2007 systematic review of the determinants of influenza and pneumococcal vaccination in the elderly, which included 14 studies from around the world, not including Canada, found the strongest predictors of pneumococcal vaccination were older age and physician recommendation [2]. Canadian studies of the knowledge and determinants of pneumococcal vaccination in older adults are lacking.

Currently, the polysaccharide pneumococcal vaccine (PPS), which covers 23 pneumococcal serotypes, is recommended and publicly funded for all adults 65 years of age and older in Canada [4] and in January 2012 the 13-valent pneumococcal conjugate vaccine was approved for use among adults over 50 years of age in Canada. The conjugate vaccine is made from purified polysaccharides of 13 different serotypes of *S. pneumonia* individually conjugated to non-toxic diphtheria cross reactive material 197 (CRM197) protein [5]. At the current time, the 13 valent conjugate vaccine is recommended and funded for Canadian infants and in some jurisdictions for adults with specific health conditions that place them at increased risk for pneumococcal infection (e.g. immunocompromised) [6]. Because pneumococcal immunization coverage rates remain low and newer conjugate vaccines may improve vaccine performance [7], the objective of this study was to understand the knowledge, attitudes and beliefs of an older population regarding pneumococcal immunization. An improved understanding of the factors influencing pneumococcal vaccine uptake among older adults could allow development of targeted interventions to promote pneumococcal immunization in hopes of improving vaccine coverage in this higher-risk age group.

## Methods

A cross-sectional survey was given to a convenience sample of adults participating in a clinical trial of seasonal influenza vaccines conducted by PCIRN (the Public Health Agency of Canada/Canadian Institutes of Health Research Influenza Research Network, http://pcirn.ca) between October 6 and November 17, 2011. Eligible participants were English or French speaking ambulatory adults 65 years of age or older, in good health or with stable health conditions, who had received the influenza vaccine within the past two years. Further, all participants lived independently or in centers providing minimal support for daily living activities. Recruitment occurred in health centers in five Canadian provinces (British Columbia, Manitoba, Ontario, Quebec and Nova Scotia). The study was approved by the following research ethics boards: University of Manitoba Bannatyne Research Ethics Board, McGill University Health Centre Biomedical Department Research Ethics Board, Hamilton Health Sciences McMaster University Research Ethics Board, IWK Research Ethics Board, Mount Sinai Hospital Research Ethics Board, Ottawa Hospital Research Ethics Board, Centre Hospitalier Universitaire de Quèbec comitè d’èthique, and the University of British Columbia, Children’s & Women’s Health Centre of BC Research Ethics Board. Participants were not provided with an incentive to complete the survey and all participants provided written informed consent.

### Survey instrument

The self-administered questionnaire (supplemental information online) was based on the theory of planned behaviour [8] and the health belief model [9] and consisted of 25 questions. It measured the subject’s knowledge of pneumococcal infection and immunization (three questions), perceived risk (three questions), personal normative beliefs (three questions), attitudes towards vaccines (six questions), facilitating conditions for vaccination (three questions) and respondent characteristics (7 questions). Of the 25 questions, ten were on a six point Likert scale with options of: “strongly disagree”, “somewhat disagree”, “neither agree nor disagree”, “somewhat agree”, “strongly agree” and “I don’t know”, eight questions had the option of “yes”, “no” or “I don’t know” and seven questions targeted demographics. Demographic information collected included sex, age, ethnicity, education level, hospitalization/emergency department/walk in clinic visit in the last year, vaccination status, and approximate annual household income. A measure of frailty was assigned to each participant by a site investigator (nurse or physician) using the previously validated Canadian Study of Health and Aging Clinical Frailty Scale which is a seven point scale ranging from “very fit” to “severely frail” [10]. The survey instrument, which was previously used in a younger population of parents and pregnant women, was pilot tested on the first ten participants at each centre to ensure clarity and comprehension; however, validity and reliability were not measured. All data collected were used in the analysis. Research nurses trained in immunization reviewed all questionnaires for completeness and were available to offer participant clarification. If participants had questions regarding pneumococcal disease or the vaccine, these were addressed after the survey was completed and a pneumococcal information pamphlet was made available to all participants (supplemental information).

### Outcome measure

The outcome variable used was self-reported receipt of the pneumococcal vaccine. All study participants were asked, “Have you ever previously had the pneumonia vaccine?” Possible responses were, “yes”, “no” or “I don’t know”. Individuals indicating unknown vaccination status (n = 75, 8.75%) were excluded from the regression models. These individuals were older (29.3% > 80 years) and a larger proportion were “apparently vulnerable” (6.7%) relative to individuals who remembered their vaccination status, otherwise they were similar to the rest of the study participants.

### Statistical analysis

Data analysis was performed using SAS version 9.3 for Windows (SAS Institute Inc., Cary, NC). All variables were explored descriptively with frequencies. Due to lack of dispersion across categories, all questions on the six point Likert scale were collapsed to four categories for the purpose of model building: “strongly agree”, “somewhat agree”, “strongly or somewhat disagree” and “I don’t know” (a combination of those who responded “neither agree nor disagree” and “I don’t know”). Bi-variable analyses investigating the relationships between self-reported vaccination status and all survey questions including demographics, knowledge, attitudes and beliefs variables were performed using Pearson’s chi-square test and logistic regression.

A multi-variable logistical regression model was built to identify the variables that were associated with self-reported vaccination status. All survey variables with a significant crude relationship with vaccination status, with the exception of those identified as being collinear and those lacking the dispersion required, were included in the initial model. Demographic variables were included as potential confounders. A backward elimination process was used (alpha >0.10) to determine the best predictive model, this model was robust to stepwise and forward selection model building processes. The variable “centre”, which represented the recruitment health centre, was retained in the model to account for similarities in the outcome status between individuals from the same centre. Model fit was assessed by examining the results of the Hosmer and Lemeshow goodness-of fit test and the Deviance residuals.

## Results

### Study population

Participant demographic characteristics by vaccination status are shown in Table 1. Of the 937 eligible individuals (59.3% female), 863 completed questionnaires (response rate 92%). The mean age of participants was 73.6 years with significantly more women participating than men (59%, p < 0.01). Almost all (98%) indicated they had a regular doctor for primary care whom they had visited in the last year (96%).

**Table 1:** Description of study population overall and by vaccination status

		**Total (n = 863)**		Vaccinated* (n = 502)	
		**n**	**%**	**n**	**%**
**Sex**	Male	354	41.0	176	49.7
	Female	509	59.0	326	64.0
					
**Age category**	65- < 70	303	35.1	138	45.5
	70- < 80	407	47.2	272	66.8
	80 +	153	17.7	92	60.1
					
**Highest level of education completed**	Primary & secondary school	282	32.8	177	62.8
	College	185	21.5	114	61.6
	University	369	42.9	195	52.8
	Other	15	1.7	9	60
	Prefer not to answer	10	1.2	5	50
					
**Household income**	< $35,000	178	20.8	110	61.8
	$35,000 to $75,000	323	37.7	199	61.6
	Over $75,000	173	20.2	89	51.5
	Prefer not to answer	182	21.3	100	55.0
					
**Ethnicity**	White/Caucasian	820	95.0	475	57.9
	Asian	29	3.4	20	69.0
	Other	14	1.6	7	50.0
					
**Frailty**	Very fit	349	40.4	195	55.9
	Well	309	35.8	176	57.0
	Well, with treated co-morbid disease	177	20.5	116	65.5
	Apparently Vulnerable/Mildly frail	28	3.2	15	53.6
					
**Centre**	Ottawa	43	5.0	16	37.2
	Halifax	161	18.7	92	57.1
	Hamilton	33	3.8	9	27.3
	Montreal	155	18.0	117	75.5
	Winnipeg	101	11.7	66	65.4
	Vancouver	118	13.7	73	61.9
	Toronto	147	17.0	60	40.8
	Quebec City	105	12.2	69	65.7

*Number and percent of vaccinated individuals within each category.

Seventy-one percent (n = 615) of participants stated they had heard of a vaccine that prevents pneumonia. Of those who had heard of the vaccine, 26% (n = 157) had not been told about it by their doctor/healthcare provider. Results from the knowledge, attitudes and beliefs questions can be found in Tables 2 and 3.

**Table 2:** Distribution of responses of study population to knowledge, attitudes and belief questions by vaccination status**

	**Strongly agree**	**Somewhat agree**	**Neither agree nor disagree**	**Somewhat disagree**	**Strongly disagree**	**Don't know**
	**% (n)**	**% (n)**	**% (n)**	**% (n)**	**% (n)**	**% (n)**
**Knowledge**						
The pneumonia vaccine keeps a person from getting pneumonia*						
Vaccinated	57.6 (288)	31.2 (156)	4.4 (22)	1.0 (5)	1.0 (5)	4.8 (24)
Unvaccinated	30.4 (87)	33.2 (95)	8.4 (24)	1.4 (4)	0	26.6 (76)
**Perceived risk**						
Pneumonia is a serious disease*						
Vaccinated	91.4 (459)	6.8 (34)	0.6 (3)	0	0	1.2 (6)
Unvaccinated	83.2 (238)	14.0 (4)	0.4 (1)	0.7 (2)	0	1.8 (5)
I am at high risk for pneumonia*						
Vaccinated	18.9 (95)	25.9 (130)	20.3 (102)	10.2 (51)	7.6 (38)	17.1 (86)
Unvaccinated	8.4 (24)	24.4 (67)	22.7 (65)	12.2 (35)	9.4 (27)	23.8 (68)
A person who does NOT get the pneumonia vaccine will probably get pneumonia*						
Vaccinated	5.5 (27)	13.9 (69)	39.8 (197)	0	0.2 (1)	0.4 (2)
Unvaccinated	2.8 (8)	10.1 (29)	24.8 (71)	0.7 (2)	0.4 (1)	1.4 (4)
**Normative**						
**My doctor’s/health care provider’s recommendations are important**						
Vaccinated	89.0 (445)	9.4 (47)	0.6 (3)	0.2 (1)	0.2 (1)	0.6 (3)
Unvaccinated	84.6 (241)	13.0 (37)	1.8 (5)	0	0	0.7 (2)
**Attitudes**						
In general, vaccines are a good way to protect my heath*						
Vaccinated	93.2 (466)	(5.4) 27	0.8 (4)	0.2 (1)	0.2 (1)	0.4 (2)
Unvaccinated	83.6 (239)	12.9 (37)	101 (3)	0.7 (2)	0	1.8 (5)
I consider vaccines to be safe*						
Vaccinated	75.6 (378)	21.6 (108)	2.0 (10)	0	0.2 (1)	0.6 (3)
Unvaccinated	65.3 (186)	30.9 (88)	1.4 (4)	1.8 (5)	0	9.8 (28)
I feel that getting the pneumonia vaccine is a wise thing to do*						
Vaccinated	92.0 (460)	6.0 (30)	1.2 (6)	0.2 (1)	0.2 (1)	1.4 (7)
Unvaccinated	52.5 (150)	27.6 (79)	8.4 (24)	2.1 (6)	0.7 (2)	9.4 (27)
It is important for healthy adults over the age of 65 to get the pneumonia vaccine*						
Vaccinated	90.4 (452)	7.2 (36)	0.6 (3)	16.8 (83)	5.7 (28)	18.4 (91)
Unvaccinated	47.6 (136)	29.0 (83)	11.2 (32)	23.8 (68)	18.5 (53)	19.9 (57)
I consider the pneumonia vaccine to be safe*						
Vaccinated	76.4 (379)	18.6 (92)	2.0 (10)	0.2 (1)	0.4 (2)	2.4 (12)
Unvaccinated	40.6 (116)	25.9 (74)	11.5 (33)	1.1 (3)	0	21.0 (60)

*Questions where p < 0.05 for Pearson’s chi square test of the null hypothesis that there is no association between vaccination status and categorical response to question (based on the 4 collapsed categories as described in methods).

**Does not include responses from individuals who indicated they “Did not know” their vaccination status (n = 75).

**Table 3:** Distribution of responses to knowledge, beliefs and facilitating conditions questions by vaccination status

	**Yes**	**No**	**I don't know**
	**% (n)**	**% (n)**	**% (n)**
**Knowledge**			
I have heard about a vaccine that prevents pneumonia*			
Vaccinated	90 (452)	3.8 (19)	6.2 (31)
Unvaccinated	44.8 (128)	46.5 (133)	8.7 (25)
The pneumonia vaccine is the same as the flu vaccine*			
Vaccinated	5.3 (26)	74.9 (369)	19.9 (98)
Unvaccinated	5.2 (15)	53.9 (154)	40.9 (117)
**Normative**			
My doctor/health care provider told me about the pneumonia vaccine*			
Vaccinated	79.8 (396)	16.7 (83)	3.4 (17)
Unvaccinated	18.2 (52)	76.6 (219)	5.2 (15)
My doctor/health care provider thinks I should get the pneumonia vaccine*			
Vaccinated	77.8 (381)	7.6 (37)	14.7 (72)
Unvaccinated	14.7 (42)	23.8 (68)	61.5 (176)
My doctor/health care provider has offered me the pneumonia vaccine*			
Vaccinated	80.2 (397)	17.8 (88)	2 (10)
Unvaccinated	11.5 (33)	82.1 (235)	6.3 (18)
**Attitudes**			
It is important to use vaccines to prevent disease like pneumonia*			
Vaccinated	95.4 (472)	1 (5)	3.6 (18)
Unvaccinated	84.2 (240)	1.4 (4)	14.4 (41)
**Facilitating conditions**			
**I have a regular doctor for primary care**			
Vaccinated	98.8 (495)	1.2 (6)	NA
Unvaccinated	98.6 (282)	1.4 (4)	NA
**I have visited my primary health care provider in the last year**			
Vaccinated	97.4 (487)	2.6 (13)	NA
Unvaccinated	96.1 (274)	3.9 (11)	NA
**I have been to a hospital, emergency department or walk in clinic in the last year**			
Vaccinated	33.7 (92)	66.3 (181)	NA
Unvaccinated	38 (192)	62 (313)	NA

*Questions where p < 0.05 for Pearson’s chi square test of the null hypothesis that there is no association between vaccination status and categorical response to question.

**Does not include responses from individuals who indicated they “Did not know” their vaccination status (n = 75).

The percentage of individuals who self-identified as being in a high risk group for pneumonia varied with frailty score. Thirty seven percent of individuals who were “very fit”, 40.2% who were “well”, 45.3% who were “well with treated co-morbid disease” and 43.5% of those who were “apparently vulnerable” either strongly or somewhat agreed with the statement “I am at high risk of pneumonia”; 21.5%, 19.9%, 13.2% and 17.4% of the “very fit”, “well”, “well, with treated co-morbid disease” and “apparently vulnerable” respectively either somewhat or strongly disagreed with the same statement.

### Vaccination status and bi-variable analyses

Fifty eight percent of participants (n = 502) indicated they had previously received the pneumonia vaccine. Those with higher unadjusted odds of vaccination included: women, individuals 70–79 years old, those with less than a university education, and individuals who were well with treated co-morbid disease relative to those who were well or very fit. Further, having been offered the pneumococcal vaccine by a health care provider, having been told by a healthcare provider about the pneumonia vaccine and believing their healthcare provider thought receipt of the vaccine was a good idea were all positively associated with vaccine receipt. As these latter three variables were highly correlated, recalling having been offered the vaccine by a health care provider was chosen for use in the multi-variable models as it was the strongest predictor of vaccination status. There was variation in distribution of vaccination status by study site. Montreal had the highest percentage of participants who reported receipt of the pneumonia vaccine (75%), 10% greater than any other study site. The majority of knowledge and attitude questions had a significant crude relationship with vaccination status (Tables 2 and 3).

### Multivariable model

Variables significantly associated with receipt of vaccine in the multivariable logistic regression model are shown in Table 4. Being offered the vaccine by a health care provider had the strongest relationship with vaccine receipt. Other variables that remained in the multivariable model included having heard of the vaccine, and strongly agreeing that it is important for adults older than 65 years to be vaccinated and age category.

**Table 4:** **Variables associated with self-reported pneumococcal vaccination status in multivariable analysis**^**a, b**^

	**Unadjusted OR**	**95% CI**		**Adjusted OR**	**95% CI**	
**I have heard about a vaccine that prevents pneumonia**						
No	Reference			Reference		
Don’t know	8.68	4.25	17.71	3.25	1.06	9.93
Yes	24.71	14.7	41.53	10.14	4.74	21.68
**The pneumonia vaccine is the same as the flu vaccine**						
Yes	Reference			Reference		
Don’t know	0.48	0.24	0.96	1.36	0.45	4.18
No	1.38	0.71	2.68	2.75	0.94	8.01
**My doctor/health care provider has offered me the pneumonia vaccine**						
No	Reference			Reference		
Don’t know	1.48	0.66	3.34	1.37	0.49	3.84
Yes	32.13	20.87	49.45	23.38	13.44	40.69
**It is important for healthy adults over the age of 65 to get the pneumonia vaccine**						
I don’t know	Reference			Reference		
Strongly agree	19.61	9.77	39.37	3.33	1.2	9.2
Somewhat agree	2.56	1.18	5.56	0.88	0.28	2.77
Somewhat/Strong disagree	1.48	0.27	7.98	1.08	0.08	15.55
**Education**						
University	Reference			Reference		
College/Other	1.33	0.92	1.92	0.78	0.41	1.46
Primary/secondary	1.6	1.13	2.25	1.49	0.82	2.72
**Frailty**						
Very Fit	Reference			Reference		
Well	1.22	0.88	1.69	1.59	0.9	2.81
Well, with treated co-morbid disease	1.87	1.24	2.82	1.96	0.94	4.09
Apparently vulnerable	1.3	0.54	3.15	1.77	0.31	9.95
**Age**						
65- < 70	Reference			Reference		
70- < 80	2.67	1.93	3.69	4.02	2.3	7.03
80 +	2.43	1.56	3.78	3.67	1.66	8.12

^a^excludes participants who indicated “I don’t know” to having received the pneumococcal vaccine (n = 75) and with those with missing data for explanatory variables (n = 35).

^b^Centre is in model to adjust for similarities between participants from the same centre.

## Discussion

Fifty-eight percent of study participants recalled receiving the pneumococcal vaccine, a result that is 20% higher than the findings of the 2006 National Immunization Coverage Survey (NICS) which found only 39% of Canadians ≥ 65 years of age recalled being immunized against pneumococcal disease [3]. This could reflect a true increased coverage among older adults in Canada, or it could be reflective of differences in our study population, which was highly compliant with annual influenza vaccination. In 2006, the national target for pneumococcal vaccine coverage was 80% among older adults. Although our results are still well below this level, the data suggest an increasing trend.

Vaccine provision by a healthcare provider is consistently found to be one of the strongest independent predictors of pneumococcal and influenza vaccine receipt among the elderly [11-14], a finding reproduced in this study. Although most participants had access to a health care provider (97% had seen their primary care provider in the past year), and therefore the opportunity to be offered the vaccine, only 52% remembered their health care provider offering them the pneumococcal vaccine at any time. This is important as approximately 9 out of 10 participants who indicated they had been offered the pneumococcal vaccine by a healthcare provider in the past also remembered being vaccinated compared to 3 out of 10 participants who did not recall being offered the vaccine by a healthcare provider. Healthcare providers should routinely offer to immunize their older patients with the pneumococcal vaccine. They have an essential role in educating their patients about the risks of IPD. In a recent meta-analysis, clinician reminders and education and patient outreach involving personal contact were all identified as factors that have been observed to improve coverage of pneumococcal vaccination among community dwelling adults [15].

Uncertainty about the effectiveness of pneumococcal vaccines among physicians may help to explain the apparent lack of advocacy for immunization. Some clinical trials and recent large observational studies have found that the polysaccharide vaccine is associated with reduced risk of pneumococcal bacteremia, presumptive pneumococcal pneumonia and both pneumococcal and all-cause community acquired pneumonia [16-19] however, a meta-analysis of clinical trials investigating the efficacy of PPS in adults found higher quality trials failed to find a protective effect [16]. A recent nested case–control study found the effectiveness of the vaccine higher for females compared to males (68% vs. 34% respectively) [20]. These conflicting results make it difficult to determine the true efficacy of the vaccine. It is possible, that if strong evidence of improved performance of the conjugate vaccine relative to PPS in adult populations is found, clinicians may be more motivated to provide recommendations to their adult patients. Other barriers to physicians immunizing with PPS that have been previously identified include other urgent concerns dominating office visits, rarity of pneumonia in their daily practice, not being able to determine vaccine status and lack of knowledge regarding the possibility of revaccination [21-23].

Consistent with previous findings from the United States [14], Spain [24] and Sweden [25], individuals 70 years of age or older had a significantly higher probability of having been vaccinated relative to participants less than 70 years of age. Also consistent with previous findings, participants who were “well, with treated co-morbidities” had a higher probability of vaccine receipt relative to those who were deemed “very fit” on the frailty scale [25,26]. This observation may be partially explained by self-perceived risk of pneumococcal infection. Individuals who were deemed “very fit” or “well” were significantly more likely to strongly or somewhat disagree to the statement “I am at high risk for pneumonia” relative to those who were deemed “well, with treated-co-morbid disease” or “apparently vulnerable” (20.2% vs 12.2%). A 2007 meta-analysis of the relationship between risk perception and vaccination behaviour among adults found that an individual’s perceptions of risk, and perceived severity of the disease are both associated with vaccination behaviour [27]. This suggests that one area of intervention to increase vaccine uptake could include education programs for individuals in their late 60’s and who are apparently healthy, which focus on their heightened risk of IPD and their risk of more severe outcomes due to their age.

Increasing awareness about pneumococcal vaccines and the risk of infection among older adults is needed as it has been found that not being vaccinated with PPS is directly associated with a lack of knowledge about the vaccine among adults 65 years of age of older [14]. We found that having heard of the vaccine and knowing that the pneumococcal vaccine was different than the influenza vaccine were associated with a higher probability of recalling vaccination. Although not surprising, these findings are an important reminder that the older adult population should be educated in an ongoing fashion regarding the availability of vaccinations that can be accessed to maintain good health. Among the vaccine-accepting population recruited for this study, 20% indicated they had not heard of the pneumococcal vaccine despite all participants being eligible to receive the vaccine free of charge. The proportion of individuals lacking knowledge of the vaccine is likely higher among the general older adult population of Canada.

Because a recommendation from a healthcare provider is the strongest predictor of vaccination, information on the pneumococcal vaccine would likely be well-received from such a source and healthcare providers will be the key source for most people. However, other sources of communication may also be important. Approximately 26% of participants in our study who had heard of the pneumococcal vaccine said their healthcare provider did not tell them about it or offer it to them, indicating that these participants accessed their information differently. Of interest is the developing role of pharmacists in providing vaccinations in Canada. Pharmacists are, or will soon be licensed to vaccinate in six provinces and are another potential route of informing the population about the vaccines recommended and available to them [28].

Individuals participating in this study had a history of vaccination, since they had all received an influenza vaccine in the prior two years. They were ambulatory adults 65 years of age or older, in good health or with stable health conditions. With this in mind, these results may not be generalizable to all older adults in Canada. Even in this highly motivated population, the uptake was lower than the national target indicating room for improvement. The outcomes of vaccination status, and having been offered the pneumococcal vaccine by a health care provider were both based on self-report and thus subject to recall bias. Finally, this is an analysis of cross-sectional data so it is not possible to determine temporality. It is not clear if participants heard of the vaccine because they were vaccinated or were vaccinated because they had heard of the vaccine. Although temporality cannot be established, it remains valuable to consider the strongest relationships identified and the role these variables may play in increasing vaccination against IPD.

## Conclusions

Coverage with PPS among older adults in Canada remains low. The results of this study indicate that investing resources to educate healthy seniors about their risk of IPD could increase coverage with PPS. The largest gains in coverage would likely be achieved through improved education of patients and physicians as well as recommendations to vaccinate, and provision of vaccine by health care providers.

## Competing interests

AS: None. JAB: None. MD: None. CLC: None. BLC: None. ML: None. ER: Has received payments for lectures and participation in advisory’s for Pfizer and Merck. SH: Acts as a co-investigator in clinical trials and surveillance studies funded by Pfizer. No financial interests. JM: Participated on advisory boards and in the preparation of educational materials for Merck. DWS: Participated on an advisory board on pneumococcal vaccines (2010) sponsored by Pfizer and holds an unrelated investigator-initiated grant from Pfizer. SAM: Holds an investigator initiated research grant from Pfizer and acts as an investigator for studies funded by Pfizer and Merck. BW: In the last 5 years has helped Wyeth (Pfizer) develop CME teaching tools related to pneumococcal vaccination. Has also received honoraria from Pfizer for talks related to vaccine use in adults (including pneumococcal vaccine) and vaccine science in general. Pfizer had no input regarding the content of these talks other than the suggested topic.

## Authors’ contributions

AS participated in the study design, carried out statistical analysis, and wrote the manuscript. JAB designed and supervised the study and data collection, provided direct supervision of AS and revised the manuscript. MD participated in the study design, implementation and data collection at the Quebec City site. CLC participated in the study design, implementation and data collection at the Ottawa site. BLC participated in the study design, implementation and data collection at the Toronto site. ML participated in the study design, implementation and data collection at the Hamilton site. ER participated in the study design, implementation and data collection at the Winnipeg site. SH participated in the study design, implementation and data collection at the Halifax site. JM participated in the study design, implementation and data collection at the Vancouver site. DWS participated in the study design, implementation and data collection at the Vancouver site. SAM participated in the study design, implementation and data collection at the Ottawa site Halifax. BW participated in the study design, implementation and data collection at the Montreal site. All authors read and approved the final manuscript.

## Pre-publication history

The pre-publication history for this paper can be accessed here:

http://www.biomedcentral.com/1471-2458/14/442/prepub

## References

[B1] Public Health Agency of Canada Vaccine Preventable Diseases: Invasive Pneumococcal Disease 2011 http://www.phac-aspc.gc.ca/im/vpd-mev/index-eng.php

[B2] Kohlhammer Y Schnoor M Schwartz M Raspe H Schäfer T Determinants of influenza and pneumococcal vaccination in elderly people: a systematic review Public Health 2007 121 742 51 10.1016/j.puhe.2007.02.011 17572457

[B3] Group ER Canadian Adult National Immunization Coverage (Adult NICS) Survey 2006

[B4] Public Health Agency of Canada (PHAC) Canadian Immunization Guide - Public Health Agency of Canada 2007

[B5] Pfizer Canada Inc Prevnar 13 (Pneumococcal 13-valent Conjugate Vaccine) Product Monograph 2014 Kirkland

[B6] National Advisory Committee on Immunization - Public Health Agency of Canada Update on the use of conjugate pneumococcal vaccines in childhood Canada Commun Dis Rep 2010 10.14745/ccdr.v36i00a12 PMC6802447 31697280

[B7] Frenck RW Yeh S The development of 13-valent pneumococcal conjugate vaccine and its possible use in adults Expert Opin Biol Ther 2012 12 63 77 10.1517/14712598.2012.636348 22074323

[B8] Ajzen I The theory of planned behavior Organ Behav Hum Decis Process 1991 50 179 211 10.1016/0749-5978(91)90020-T

[B9] Janz NK Becker MH The health belief model: a decade later Heal Educ Behav 1984 11 1 47 10.1177/109019818401100101 6392204

[B10] Rockwood K Song X MacKnight C Bergman H Hogan DB McDowell I Mitnitski A A global clinical measure of fitness and frailty in elderly people CMAJ 2005 173 489 95 10.1503/cmaj.050051 16129869 PMC1188185

[B11] Nichol KL Mac Donald R, Hauge M Factors associated with influenza and pneumococcal vaccination behavior among high-risk adults J Gen Intern Med 1996 11 673 7 10.1007/BF02600158 9120653

[B12] Evans MR Watson P Why do older people not get immunised against influenza? a community survey Vaccine 2003 21 2421 2427 10.1016/S0264-410X(03)00059-8 12744874

[B13] Nowalk MP Zimmerman RK Shen S Jewell IK Raymund M Barriers to pneumococcal and influenza vaccination in older community-dwelling adults (2000–2001) J Am Geriatr Soc 2004 52 25 30 10.1111/j.1532-5415.2004.52006.x 14687311

[B14] Ehresmann KR Ramesh A Como-Sabetti K Peterson DC Whitney CG Moore KA Factors associated with self-reported pneumococcal immunization among adults 65 years of age or older in the Minneapolis-St. Paul metropolitan area Prev Med (Baltim) 2001 32 409 15 10.1006/pmed.2001.0839 11330990

[B15] Lau D Hu J Majumdar SR Storie DA Rees SE Johnson JA Interventions to improve influenza and pneumococcal vaccination rates among community-dwelling adults: a systematic review and meta-analysis Ann Fam Med 2012 10 538 46 10.1370/afm.1405 23149531 PMC3495928

[B16] Huss A Scott P Stuck AE Trotter C Egger M Efficacy of pneumococcal vaccination in adults: a meta-analysis CMAJ 2009 180 48 58 10.1503/cmaj.080734 19124790 PMC2612051

[B17] Wagner C Popp W Posch M Vlasich C Rosenberger-Spitzy A Impact of pneumococcal vaccination on morbidity and mortality of geriatic patients: a case-controlled study Gerontology 2003 49 4 246 250 10.1159/000070405 12792160

[B18] Sharpiro E Berg A Austrian R Schroeder D Parcells V Margolis A Adair R JD C The protective efficacy of polyvalent pneumococcal polysaccharide vaccine N Engl J Med 1991 325 21 1453 60 10.1056/NEJM199111213252101 1944423

[B19] Ochoa-Gondar O Vila-Corcoles A Rodriguez-Blanco T Gomez-Bertomeu F Figuerola-Massana E Raga-Luria X Hospital-Guardiola I Effectiveness of the 23-valent pneumococcal polysaccharide vaccine against community-acquired pneumonia in the general population aged ≥ 60 years: 3 years of follow-up in the CAPAMIS study Clin Infect Dis 2014 58 909 17 10.1093/cid/ciu002 24532544

[B20] Wiemken TL Carrico RM Klein SL Jonsson CB Peyrani P Kelley RR Aliberti S Blasi F Fernandez-Gonzalez R Lopardo G Ramirez JA The effectiveness of the polysaccharide pneumococcal vaccine for the prevention of hospitalizations due to Streptococcus pneumoniae community-acquired pneumonia in the elderly differs between the sexes: results from the community-acquired pneumonia organi. Vaccine 2014 32 2198 203 10.1016/j.vaccine.2014.02.048 24613522

[B21] Mieczkowski TA Wilson SA Adult pneumococcal vaccination: a review of physician and patient barriers Vaccine 2002 20 1383 1392 10.1016/S0264-410X(01)00463-7 11818157

[B22] Szilagyi PG Shone LP Barth R Kouides RW Long C Humiston SG Jennings J Bennett NM Physician practices and attitudes regarding adult immunizations Prev Med (Baltim) 2005 40 152 61 10.1016/j.ypmed.2004.05.010 15533524

[B23] Mui LWH Chan AYS Lee A Lee J Cross-sectional study on attitudes among general practitioners towards pneumococcal vaccination for middle-aged and elderly population in Hong Kong PLoS One 2013 8 e78210 10.1371/journal.pone.0078210 24223775 PMC3818320

[B24] Sintes X Nebot M Izquierdo C Ruiz L Dominguez A Bayas JM Vera I Carratala J Sousa D Factors associated with pneumococcal and influenza vaccination in hospitalized people aged 65 years Epidemiol Infect 2011 139 666 673 10.1017/S0950268810001846 20696084

[B25] Christenson B Lundbergh P Comparison between cohorts vaccinated and unvaccinated against influenza and pneumococcal infection Epidemiol Infect 2002 129 515 24 10.1017/S095026880200780X 12558334 PMC2869913

[B26] Lu P Nuorti JP Pneumococcal polysaccharide vaccination among adults aged 65 years and older, U.S., 1989–2008 Am J Prev Med 2010 39 287 95 10.1016/j.amepre.2010.06.004 20837278

[B27] Brewer NT Chapman GB Gibbons FX Gerrard M McCaul KD Weinstein ND Meta-analysis of the relationship between risk perception and health behavior: The example of vaccination Heal Psychol 2007 26 136 45 10.1037/0278-6133.26.2.136 17385964

[B28] Wong-Beringer A Brodetsky E Quist R Pneumococcal vaccination in hospitalized elderly patients: role of the pharmacist Pharmacotherapy 2003 23 199 208 10.1592/phco.23.2.199.32085 12587809

